# Enable, engage, and innovate for quality

**DOI:** 10.1136/bmj.p2396

**Published:** 2023-12-11

**Authors:** Khumbize Kandodo Chiponda, Lia Tadesse, Selina Dussey, Juan-Pablo Uribe, Anshu Banerjee

**Affiliations:** 1 Ministry of Health, Lilongwe, Malawi; 2 Ministry of Health, Addis Ababa, Ethiopia; 3 Ministry of Health, Accra, Ghana; 4 World Bank, Washington, DC, USA; 5 Department Maternal Newborn Child Adolescent Health and Ageing, World Health Organization, Geneva, Switzerland

## Abstract

An approach to consistently deliver quality healthcare to everyone, everywhere

Quality healthcare is fundamental to the right to health and its delivery at all times.^1^ People are healthier and are living longer than ever before, but poor quality care is still causing preventable deaths and unacceptable inequities, especially in low and middle income countries. Despite widespread recognition of the importance of quality health services, improvement efforts continue to struggle against persistent gaps in financing, workforce, and healthcare infrastructure, alongside the overlapping global challenges from pandemics, conflicts, and climate change.^2^ ^3^ As the covid-19 pandemic showed, quality matters even more for population health during a crisis.^4^

Health systems in low and middle income countries operate in different contexts and possess different capacities. Despite these differences, quality care can be sustained and scaled up if it is championed by consistent leadership and supported by an enabling environment that encourages engagement of all actors in continuous improvement and stimulates a culture of innovation.

The drive for quality in health starts at the top. Leadership has a pivotal role in setting the vision, values, and priorities that drive efforts to increase the quality of care.^5^ However, leadership must be consistent and go beyond a political statement. Since 2015, national quality management policies and strategies in Bangladesh, Ethiopia, Ghana, and Malawi have guided these countries’ actions to improve quality across all levels of healthcare. Newly established quality of care governance mechanisms are leading, supporting, monitoring, and evaluating the implementation of quality efforts. These governance mechanisms for healthcare quality will have maximum effect only if all health programmes and relevant stakeholders participate to co-create and implement quality driven solutions, demonstrate accountability for results, and scale up best practices and innovations.

Quality healthcare depends on the availability of resources, both human and financial, to support ongoing training, technological advancements, and infrastructure development. While ensuring an adequate level of resources for health remains a considerable challenge for most low and middle income countries, an enabling environment for quality can still encourage and strengthen the capacity of the health workforce to deliver quality care. Training and continuous professional development, effective leadership, teamwork, and a culture of learning and accountability enable healthcare providers to embrace change for quality, share best practices, learn from mistakes, and improve health outcomes. Examples such as Ghana’s networks of care approach, integrated within primary healthcare, show that relationship building and shared learning among health providers and health service organisations improves quality.^6^

An enabling environment for quality creates the space to engage and maximise the contribution of traditional stakeholders such as professional associations, academia, and civil society, as well as new ones, such as the private sector. Growing experience is showing that private sector provision has the potential to address some of the challenges faced by health systems in low and middle income countries. Country efforts to engage the private sector have also shown a pressing need for new governance and regulatory tools and mechanisms to facilitate such engagement.^7^

## People centred care

We know that good quality care is people centred, responds to users’ expectations, and fulfils their right to health. Community engagement mechanisms create the basis for cultivating trust and collaboration between providers and the communities they serve. In the past decade we have witnessed a surge in mechanisms for community and user engagement. These mechanisms, often driven by the health system, aim to ensure users’ participation and demonstrate accountability of health services. Examples include the community scorecards in Ethiopia and Ghana, which are co-developed and jointly implemented with communities.^8^ ^9^

However, the rapid shift in communication and information sharing brought by technology and social media is profoundly altering users’ expectations for quality and use of services. The future of quality will be defined by people’s voices shared through platforms that are not led or managed by health providers. The ability of health systems and providers to deliver quality care will be defined by their ability to listen to people’s expectations and needs, along with the ability to demonstrate accountability, share the right information, and correct any misconceptions that undermine public trust.

Learning and innovation tailored to the specific countries’ needs and context stimulate and support continuous improvement across the entire healthcare system. Digital solutions such as those related to commodities supply management or patient counselling and tracking, can help tackle persistent challenges of quality and access to services.^10^ Proactive research and development will help countries to identify the most effective innovations for delivering quality care in their own contexts.

Quality healthcare is not a “nice to have” or a one-off project with a start and end date but must be consistently delivered to everyone, everywhere, starting from primary health services to the highest levels of care.^11^ To be most effective, current and future investments in health have to be informed by quality gaps, address quality needs, and contribute towards developing an enabling environment for quality.

## Notes

Provenance and peer review: Commissioned; not externally peer reviewed.

This article is part of a collection proposed by the World Health Organization and the World Bank and commissioned by *The BMJ*. *The BMJ* peer reviewed, edited, and made the decision to publish these articles. Article handling fees are funded by the Bill & Melinda Gates Foundation. Jennifer Rasanathan, Juan Franco, and Emma Veitch edited this collection for *The BMJ*. Regina Kamoga was the patient editor.
